# The Two Sides of YY1 in Cancer: A Friend and a Foe

**DOI:** 10.3389/fonc.2019.01230

**Published:** 2019-11-20

**Authors:** Sailu Sarvagalla, Srinivasa Prasad Kolapalli, Sivakumar Vallabhapurapu

**Affiliations:** Division of Biology, Indian Institute of Science Education and Research Tirupati, Tirupati, India

**Keywords:** YY1, transcriptional activation, transcriptional repression, tumor suppressor, tumor promoter, protein-protein interaction, miRNA and small molecule inhibitor

## Abstract

Yin Yang 1 (YY1), a dual function transcription factor, is known to regulate transcriptional activation and repression of many genes associated with multiple cellular processes including cellular differentiation, DNA repair, autophagy, cell survival vs. apoptosis, and cell division. Owing to its role in processes that upon deregulation are linked to malignant transformation, YY1 has been implicated as a major driver of many cancers. While a large body of evidence supports the role of YY1 as a tumor promoter, recent reports indicated that YY1 also functions as a tumor suppressor. The mechanism by which YY1 brings out opposing outcome in tumor growth vs. suppression is not completely clear and some of the recent reports have provided significant insight into this. Likewise, the mechanism by which YY1 functions both as a transcriptional activator and repressor is not completely clear. It is likely that the proteins with which YY1 interacts might determine its function as an activator or repressor of transcription as well as its role as a tumor suppressor or promoter. Hence, a collection of YY1-protein interactions in the context of different cancers would help us gain an insight into how YY1 promotes or suppresses cancers. This review focuses on the YY1 interacting partners and its target genes in different cancer models. Finally, we discuss the possibility of therapeutically targeting the YY1 in cancers where it functions as a tumor promoter.

## Introduction

Yin Yang 1 (YY1) (also called as ∂ transcription factor, nuclear factor-E1 (NF-E1), INO80 complex subunit S and upstream control region binding protein) is an evolutionarily conserved C_2_H_2_ zinc finger containing multi domain transcription factor with several functions (1–9). It was first identified in 1991 by several independent research groups as DNA binding protein, and was shown to act as a transcriptional activator/repressor of adeno-associated virus (AAV) P5 promoter, and other genes (1–7). In the presence/absence of oncoprotein E1A, YY1 acts as P5 promoter activator/repressor, respectively. Hence, the protein was named as Yin Yang 1 (YY1) from the Chinese word “Yin,” for repression, and “Yang” for activation.

YY1 is ubiquitously expressed in human tissues and controls various cellular mechanisms including transcriptional regulation (activation/repression), cell proliferation, DNA repair, chromatin modeling, apoptosis, autophagy, X-chromosome inactivation, recruitment of Polycomb Group (PcG) proteins and epigenetic modifications (1, 2, 10–13). YY1 has also been implicated in B cell maturation, development and immunoglobin class switch recombination (14, 15). Depending on its interacting partners (Protein-Protein Interaction-PPI), promoter environment and chromatin structure, YY1 was shown to regulate several genes that are involved in various homeostatic processes and diseases including cancer (16, 17). Typically, YY1 regulate genes either by direct binding to the corresponding gene promoters or indirectly through association with chromatin remodeling proteins and histone modifiers (18, 19). Involvement of YY1 in the regulation of genes that are directly linked to malignant transformation indicated that YY1 might play a key role in many cancers, and indeed YY1 has been reported to be highly expressed in many cancers and is essential for cancer progression (20, 21). However, surprisingly, YY1 has also been reported to play a tumor suppressor role particularly in the case of pancreatic cancer (22). In case of breast cancer and few other cancers, YY1 has been reported to play both a tumor promoter and a suppressor role (23, 24), which is quite puzzling. Perhaps the cellular context, differential interaction with other pro-tumorigenic vs. tumor suppressive molecules may dictate the behavior of YY1 as a tumor promoter vs. tumor suppressor. Therefore, better understanding of YY1 protein structure, function, and its altered protein-protein interactions (PPIs) in various cancers would not only help understand the role of YY1 in cancer but also in the design and development of novel therapeutic strategies. In this review, we describe YY1 structure, function, and its protein-protein interactions (PPIs) in the context of gene regulation and various cancers. Additionally, we discuss the reported YY1 inhibitors and their role in cancer therapeutics.

## YY1 Sequence

**Figure 1:**
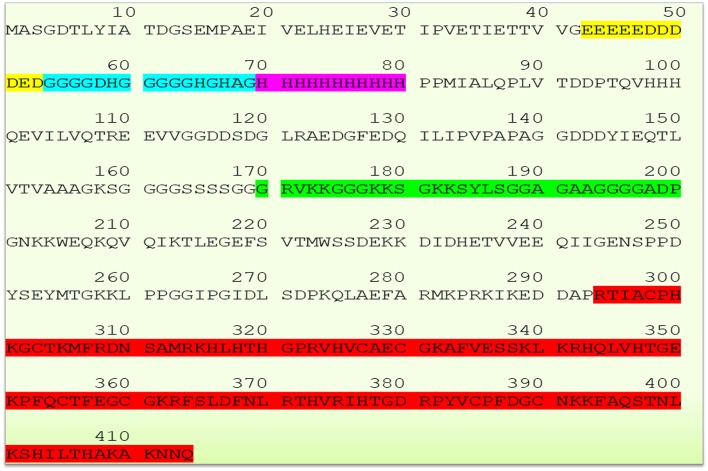
Schematic representation of YY1 sequence and its highlighted features (Glutamic and Aspartic acid rich region represented in yellow color, Glycine rich region in blue color, histidine rich region in magenta color, transcriptional repressive region in green color, and zinc finger sequence in red color, respectively). YY1 protein sequence was retrieved from UniProt database (UniProt entry ID: P25490).

The human YY1 is a highly conserved 414 amino acids protein with multiple domains. Its sequence is highly homologs and shares 99.8 and 90.3% identity with mouse and *xenopus laevis*, respectively (2, 5, 20, 25). The N-terminal region of YY1 contains transcriptional activation domain (amino acid sequence 1–100) with distinguished features. Amino acid sequence from 43 to 53 comprises 11 uninterrupted acidic residues (Glutamic & Aspartic acids) whereas amino acid sequence from 70 to 80 contains 11 successive histidine residues; and these two regions are connected by a glycine rich (amino acid sequence 54–69) region. Due to its acidic nature, the N-terminal region of YY1 has been reported to be involved in electrostatic interactions particularly with positively charged proteins (2). While, the histidine tract within the N-terminal region of YY1 has been described to be involved in nuclear localization (26), the central region of YY1 (amino acid sequence from 170 to 200), and the sequence near to the carboxyl terminal region (amino acids 333–397) were reported to be involved in transcriptional repression (5, 27). An additional region within the central domain (PHR, amino acids 205–226) mediates the interaction with homeobox Hox proteins, which plays important role in development. Indeed, YY1 is essential for mammalian development as evidenced by the embryonic lethality in YY1 homozygous knockout mice (28). The C- terminal region of YY1 (amino acid sequence 294–414) contains four C_2_H_2_-type zinc-finger motif that mediates DNA binding. Thus, YY1 uses these domains to interact and recruit various transcriptional regulators and proteins including transcription factor IIB, RNA polymerase II, poly (ADP-ribosyl) polymerase, p300, c-Myc, pRb, HDACs, CREB, FK506-binding protein, Sp1, NOTCH, YY1-associated factor 2, ATF6, GATA3 C/EBPb and etc. (20, 29). The sequence of YY1 and its highlighted features are represented in Figure 1.

## YY1 Structure

Although, YY1 has been implicated in many aspects of mammalian biology, little is known about its structure. Since YY1 seems to be an intrinsically disordered protein (IDP) that shows relatively less globularity & low compactness, high flexibility, and dynamic behavior under physiological conditions (30, 31), it might be difficult to get full crystal structure for this protein. It has been proposed that intrinsically disordered proteins frequently act as “hubs” in protein-protein interaction networks; and thereby by regulate the signaling pathways associated with various crucial cellular processes including transcription, translation, and cell cycle etc. (32). Accordingly, YY1 has been shown to indulge in various cellular processes with the help of multiple binding partners. Analysis of YY1 structure using various methods together with bioinformatics approaches (30) revealed that the N-terminus of YY1 contains an intrinsically disordered non-compact structure and lacks a well-defined tertiary structure. While the full length crystal structure of YY1 is not available, co-crystal structure of YY1 zinc fingers (amino acids 293–414) in association with initiation element of AVV P5 promoter (20-bp oligonucleotide) has been reported at 2.5 Å resolution (PDB ID: 1UBD) (33). Another region within YY1 whose structure is relatively well-documented is the so called REPO domain (named for its function in recruitment of Polycomb proteins (34), spanning amino acids 201–226. This 26aa sequence was shown to be sufficient for transcriptional repression, recruitment of polycomb group (PcG) proteins to DNA and to cause methylation of lysine 27 on histone H3 (35). Further, crystal structure of this motif in complex with the four human malignant brain tumor (4MBT) domain containing protein MBTD1 has been shown to form anti-parallel β-sheets (36) and has been reported to regulate polycomb response element containing genes (36). Therefore, resolving full length YY1 structure using experimental methods (i.e., X-ray and NMR) or computational methods (i.e., homology modeling and threading) could provide significant insights related to its function and molecular mechanism.

## YY1 Protein-Protein Interactions (PPIs) Implicated in Gene Regulation

YY1 was reported as an intrinsically disordered protein with multiple binding partners and varied localizations (30). Analysis of curated PPI databases such as STRING (https://string-db.org/), GPS-Prot (http://gpsprot.org/), BIOGRID (https://thebiogrid.org/), MINT (https://mint.bio.uniroma2.it/) and HINT (http://hint.yulab.org/) revealed that YY1 interacts with several proteins and cofactors and reported to regulate transcription, chromatin organization, histone acetylation & deacetylation, epigenetic modification, DNA repair, ubiquitination, apoptosis, cell proliferation and tumor suppression/promotion. In this review, we described YY1 PPIs and their functional implications in tumor growth and suppression.

## Role of YY1 in Transcriptional Regulation

As mentioned before, depending on its interacting partners, YY1 acts as both transcriptional activator and repressor. Initially, Shi et al. (25) reported that YY1 interacts and modulates the E1A protein to control AAV P5 promoter activity. In the absence of E1A protein, YY1 repressed the P5 promoter activity whereas in the presence of it YY1 caused transcriptional activation. However, the molecular mechanism by which binding of E1A converts YY1 to transcriptional activator was not clear from this study. Subsequently, Lee et al. (18) showed that E1A recruits transcriptional co-activator p300 to YY1 and thereby leads to transcriptional activation. Besides, recent evidence reports that YY1 acetylation /deacetylation contributes to its transcriptional activity (19). Acetylation of YY1 through p300 leads to transcriptional activation, whereas deacetylation of YY1 through HDACs leads to transcriptional repression.

Owing to its role in activation and repression of genes, it is likely that YY1 interacts with a wide range of factors that would govern its function as transcriptional activator/repressor. Indeed, the activity of YY1 was shown to be controlled by a wide variety of protein-protein interactions including basal transcription machinery proteins (e.g., TATA binding protein, TFIIB, TBP, TAF55, RNA polymerase II etc.) sequence-specific DNA-binding transcriptional activators (e.g., SpI, c-Myc, ATF/CREB, C/EBP), and various transcriptional coregulatory molecules (e.g., E1A, TAFII55, p300, CREB-binding protein (CBP), HDAC1, HDAC2, and HDAC3) (18, 19).

YY1 has been shown to regulate the expression of many genes either positively or negatively in the context of various cellular processes (20, 29). As a transcriptional activator, YY1 positively regulates many genes. One of the mechanisms by which YY1 regulates gene expression is by competing and preventing the binding of repressors to the gene promoters. For example, Melanie et al. (37) reported that YY1 directly binds to Xist 5′ region and triggers the activity of Xist promoter. In this case, YY1 competes with Xist repressor REX1 for binding to the Xist 5′ region. However, the specific nature of the transcriptional activating complex nucleated by YY1 on Xist promoter is not clear from this study. Nevertheless, in many other cases, specific YY1 interacting partners involved in transcriptional co-activation have been reported (38). For instance, ATF6, which plays a key role during ER stress and induces glucose-regulated protein (grp) genes that encode ER chaperones, has been shown to interact with YY1 and that YY1 interaction with ATF6 enhances its transcriptional activity to induce “grp” genes (39). Importantly, a recent report described that INO80, a chromatin remodeling enzyme is an essential transcriptional coactivator for YY1 (8). YY1 interacts with INO80 and recruits it to its target genes to induce their expression. Interestingly, INO80 not only acts as a co-activator but also helps YY1 to gain access and bind to target promoter sites (8). One of the genes that INO80-YY1 complex activates is CDC6, that plays a role in DNA replication and cell division (9). In addition, the INO80-YY1 complex has been shown to regulate genomic stability and participate in homologous recombination mediated DNA repair (8, 9). Furthermore, YY1 reported to be involved in transcriptional initiation by interacting with Sp1. YY1-Sp1 interaction has been shown to initiate transcription of Mu Opioid Receptor (MOR) gene in Human Lymphocytes (40). Since the MOR activation is linked to suppression of lymphocyte proliferation, YY1-Sp1 interaction might suppress lymphocyte proliferation via MOR upregulation.

Many reports indicated that YY1 inhibits transcription of several genes by interacting with various proteins. For instance, YY1 coordinates with NF-κB to repress Bim, which is a proapoptotic member of the Bcl2 family. While the NF-κB signaling has been shown to be involved in the induced expression of YY1 (41), it is of interest to note that, in multiple myeloma, YY1 physically interacts with the canonical NF-κB RelA and that the YY1-RelA complex represses Bim promoter (41). This observation is of significant interest because RelA, which is largely appreciated as a transcriptional activator, has been shown to function as a transcriptional repressor on Bim promoter upon forming a complex with YY1. Owing to the pro-apoptotic function of Bim, its repression by the YY1-RelA complex has been shown to play an important role in the survival of multiple myeloma cells (41). Further, other members of the NF-κB family including RelB and cRel were shown to transcriptionally induce the expression of YY1 in response to TLR3 and that YY1 downstream of TLR3 was shown to bind to IFN-β promoter and repress it by preventing the binding of IRF7 to IFN-β promoter (42). The repressive function of YY1 downstream of TLR3 seems to play an important role in fine-tuning the amount and duration of IFN-β expression (42). It is however, not clear whether in this case YY1 forms a transcriptional repressive complex with NF-κB. Interestingly, while the YY1-RelA complex was shown to function as a transcriptional repressor (41), a complex between RelB and YY1 was shown to function as a transcriptional activator in glioblastoma multiforme (GBM) cells and promotes GBM growth (43). Likewise, YY1 interacts with a large number of factors to regulate various genes. A list of YY1 interacting proteins and their role in gene regulation is provided in Table 1.

**Table 1:** YY1 protein-protein interacting partners and their role in transcriptional activation/repression.

**S. no**.	**YY1 interacting partners**	**Functions**	**Outcome results**	**References**
**YY1 partners involved in transcriptional repression**				
1	HDAC1	Transcriptional repression	YY1 binds and recruits HDAC1 to the promoters of oligodendrocyte differentiation inhibitors (Tcf4 and Id4) and repress these genes	(44)
2	HDAC2	Transcriptional repression	Represses miR-500a-5p promoter in colorectal cancer	(45)
3	HDAC3	Transcriptional repression	Repression of c-Myc, p16 and Mmp-9	(46–48)
4	HDAC4	Transcriptional repression	Transcriptional repression of HOXB13 in AR negative prostate cancer cells.	(49)
5	HDAC5	Transcriptional repression	Repression of sodium calcium exchanger (Ncx1) and the brain natriuretic peptide (Bnp) genes	(50, 51)
6	SAP30	Transcriptional repression	Repression of IFN-β expression	(52, 53)
7	MECP2	Transcriptional repression	Repression of ANT1 gene	(54)
8	SMAD2 and SMAD3	Transcriptional repression	Repression of transforming growth factor (TGF-β) and bone morphogenetic protein (BMP)	(55, 56)
9	YAF2	Repressive complex	YAF2 interacts with YY1 and facilitate Polycomb group protein (PcG) recruitment.	(57)
10	LSF	Transcriptional repression	Represses the human immunodeficiency virus type 1 long terminal repeat.	(58)
11	TP53	Transcriptional repression	Inhibits p53 transcriptional activity leads to tumor progression	(59)
12	RYBP	Transcriptional repression	Represses miR-29, and negatively regulate skeletal myogenesis	(60, 61)
13	Ezh2	Transcriptional repression	Regulation of muscle gene expression and skeletal muscle differentiation	(62)
14	RelA	Transcriptional repression	YY1-RelA complex represses the pro-apoptotic gene Bim in multiple myeloma cells.	(41)
15	SFMBT2	Transcriptional repression	SFMBT2 interacts with YY1 and represses HOXB13 gene and enhances DU145 prostate cancer cell survival SFMBT2 interacts with YY1, represses MMP2, MMP3, MMP9 and inhibits LNCaP prostate cancer cell invasion and migration	(63, 64)
16	Retinoblastoma (Rb)	Transcriptional repression	Rb interacts with YY1 and inhibits YY1 DNA binding, and thereby blocks YY1-dependent transcription.	(65)
17	HOXA11	Transcriptional repression	YY1 interacts with Hoxa11 and represses Hoxa11 target genes.	(66)
18	CP2	Transcriptional repression	HXPR motif of YY1 interacts with CP2 and suppresses CP2's transcriptional activity.	(67)
**YY1 partners involved in transcriptional initiation and activation**				
19	Transcription factor IIB (TFIIB)	Transcriptional initiation	TFIIB interacts with YY1, stabilizes YY1 DNA binding and thereby facilitate transcriptional initiation.	(68)
20	SP1	Transcription initiation	YY1 and SP1 interacts and initiate expression of Mu Opioid Receptor (MOR) gene in Human Lymphocytes	(40)
21	Activator protein 2 (AP-2)	Transcriptional activation	Interaction of YY1 with AP-2 transcription factor induces ERBB2 promoter activity in breast cancer cells.	(69, 70)
22	OCT4 and BAF	Transcriptional activation	YY1 interacts with OCT4 and BAF and regulate the pluripotency network genes in mouse embryonic stem cells (mESCs)	(71)
23	INO80	Transcriptional activator and DNA repair	INO80 interacts with YY1 and function as transcriptional co-activator for YY1 target genes. Also, INO80-YY1 complex has been implicated in DNA repair.	(8, 9)
24	ATF6	Transcription activation	Transcriptional activation of glucose regulated protein (grp) genes (Grp78)	(39)
25	YY1AP1	Transcriptional activation	YY1AP1 is a co-activator of YY1, and activates transcription of stemness regulators in hepatocellular carcinoma.	(38, 72)

## YY1 in Embryogenesis and Development

While YY1 has been shown to play opposing roles in cancer, owing to its ubiquitous expression YY1 has also been implicated in embryonic development and cellular lineage differentiation. Indeed, it has been reported that homozygous deletion of YY1 in mice results in peri-implantation lethality and embryonic defects. Further, mouse embryos that were heterozygous for YY1 deletion displayed delayed development as well as neurulation defects (28). In line with this, another group in a relatively recent report have shown that complete ablation of YY1 results in failure of cytokinesis and a complete block in cell proliferation (73). Further, analysis of YY1 target genes has identified a large set of YY1 target genes with important roles in cell survival/apoptosis, proliferation, development, and differentiation indicating that YY1 coordinates a complex transcriptional network to regulate several biological processes during embryogenesis (73). In line with the data obtained from mice, human individuals with deletions or mutations in YY1 exhibited a complex syndrome that had features including behavioral abnormalities, cognitive impairment, feeding problems as well as defective intrauterine growth (74). This syndrome has been termed as the “YY1 Syndrome” caused largely by haploinsufficiency of YY1 that results in impaired Histone H3 K27 acetylation and dysregulated global transcriptional output by YY1 (74). In addition to its role in embryogenesis and tissue formation in adults, YY1 has also been shown to play important role in stem cells (71). A recent report has shown that YY1 interacts with the BAF complex and thereby contributes to proliferation and pluripotency of mouse embryonic stem cells (mESCs) (71). In line with this, deletion of YY1 or Smarca4 (a core component of BAF complex) results in a sharp decrease in pluripotent markers on mESCs (71). In addition to its role in mESCs, a recent report has shown an important role for YY1 in neural crest stem cells (NCSCs) (75). These authors, by employing a combination of metabolomics, ChiP-Seq and RNA-Seq analysis have found that YY1 controls multiple metabolic pathways in NCSCs and that conditional ablation of YY1 results in hypoplasia of all neural crest derivatives (75). Since melanocytes originate from NCSCs during development, these authors went on to show that conditional ablation of YY1 impairs melanoma tumor growth in a melanoma mouse model (75). In another recent report, it has been shown that YY1 plays a key role in trophoblast invasion at maternal–fetal interface by regulating the expression of MMP2, a key player in trophoblast invasion (76). Importantly, these authors have also found that in recurrent miscarriage (RM) patients YY1 expression is significantly decreased in their trophoblasts (76). Further it was shown that YY1 regulates the Hox transcript antisense RNA (HOTAIR) expression which in turn triggers PI3K-AKT signaling, leading to enhanced MMP2 expression and trophoblast invasion (77). Interestingly and in line with the reduced levels of YY1 in RM patients HOTAIR expression was also found to be low in RM patients, which perhaps accounts for reduced MMP2 expression in RM trophoblasts (77). Collectively, it is clear that YY1 plays a central role in embryonic development in addition to its role in cancer.

## Role of YY1 in Cancer

YY1 has been shown to be overexpressed in many cancers including lung cancer, breast cancer, ovarian cancer, colon cancer, prostate cancer, brain cancer, cervical cancer, osteosarcoma, gastric cancer, acute myeloid leukemia, B-cell and follicular lymphoma etc. (20, 21) and its expression levels largely correlated with cancer progression, metastasis, drug resistance, and poor prognosis. While YY1 has been shown to play a key role in the progression of many cancers, the mechanism by which YY1 contributes to tumor growth differs in different cancers by activating or repressing various genes in a cancer specific manner. We listed out the genes that YY1 regulate and the outcome with regards to tumor growth in Table 2. On the contrary, in addition to the opposing functions of YY1 both as a transcriptional activator and repressor, YY1 has also been shown to have two sides in cancer i.e., a tumor promoter and a tumor suppressor (96). An insight into the mechanism by which YY1 functions as a tumor promoter vs. tumor repressor would help us design novel therapeutic approaches by targeting or manipulating the function of YY1. It is likely, that the opposing roles of YY1 as tumor promoter vs. tumor suppressor depend on the cancer type and its interacting partners (Table 3) by regulating the expression or repression of several genes as well as non-coding RNAs including long non-coding RNAs (lnc) and several microRNAs (miRNAs). Of particular interest, YY1 dependent repression of miR-9, miR-29, miR-146a, and miR-489 has been shown to be pro-tumorigenic (109–112). While upregulation of some lnc-RNAs such as lncRNA-PVT1 by YY1 promotes tumor growth (90). In this section, we discuss the role of YY1 in different cancers in detail.

**Table 2:** Examples of YY1 regulated genes and their role in cancer suppression/progression.

**Cancer type**	**Gene**	**Outcome of gene regulation**	**Impact on cancer**	**References**
**Cancer suppression**				
Triple negative breast cancer	LINC00152**↓**	YY1 dependent repression of LINC00152 leads to stabilization/elevation of PTEN through E3 ligase NEDD4-1	Suppression	(78)
Breast cancer	FEN1**↓**	YY1-dependent repression of FEN1 leads to sensitization of breast cancer cells to DNA-damaging agents	Suppression	(79)
Pancreatic cancer	CDKN3**↓**	YY1 represses CDKN3 gene and thereby facilitate p21 expression	Suppression	(80)
	SOX20T**↓**	YY1 represses tumor promoting lncRNA SOX2OT, and thereby downregulates SOX2 expression in pancreatic cancer cells	Suppression	(81)
	miR30a**↓**	YY1 represses miR-30a in pancreatic cancer cells leading to enhanced autophagy and tumor suppression	Suppression	(82)
	BAX**↑**	YY1 upregulates BAX gene expression and subjects pancreatic cancer cells to apoptosis	Suppression	(83)
	MMP10**↓**	YY1 represses MMP10 and thereby suppresses metastasis of pancreatic ductal adenocarcinoma (PDAC)	Suppression	(22)
	Feline sarcoma-related (FER)	YY1 binds to FER promoter and represses its expression and inhibits invasion and migration of pancreatic cancer cells	Suppression	(84)
Lung cancer	HLJ1**↑**	YY1 coordinates with AP1 and induces expression of tumor suppressor HLJ1 leading to reduced invasiveness in lung adenocarcinoma	Suppression	(85)
**Cancer progression**				
Colon cancer	Fas**↓**	YY1 downregulates Fas expression and promotes colon cancer cell survival and growth	Progression	(86)
	DN-LEF1**↓**	YY1 represses the expression of dominant negative form of LEF1 (DN-LEF1) and potentiates Wnt signaling	Progression	(87)
	SLC22A15**↑** & AANAT	YY1 upregulates the oncogenes SLC22A15 and AANAT in colon cancer cells	Progression	(88)
	GLUT3**↑**	YY1 upregulates GLUT3 and promotes the Warburg effect	Progression	(89)
Lung cancer	lncRNA-PVT1**↑**	YY1 positively regulates lncRNA-PVT1 and thereby induce tumor cell proliferation	Progression	(90)
Prostate cancer	XAF1**↓**	YY1 represses the tumor suppressor XAF1 and thereby promotes prostate cancer growth.	Progression	(91)
Glioma	P53**↓**	Inhibits p53 expression and promotes glioma cell growth	Progression	(92)
	Pro-inflammatory cytokines**↑**	Upregulates pro-inflammatory cytokines leading to infiltration of glioma associated macrophages and tumor growth	Progression	(43)
Non-Hodgkin's lymphoma	KLF4**↑**	YY1 upregulates KLF4 expression in NHL.	Progression	(93)
Acute Myeloid Leukemia (AML)	miR-let7a**↓** MYC &BCLXL**↑**	Downstream of SDF-1α /CXCR4 axis, YY1 represses miR-let-7a and enhances MYC & BCLXL expression in AML cells and promotes their survival and proliferation	Tumor progression	(94)
Breast cancer	P27 **↓**	YY1 represses p27 expression and thereby promotes tumor formation.	Progression	(24)
	ERBB2**↑**	Coordinated function of YY1 and AP2 induces the oncogene ERBB2 in breast cancer cells and promote tumor growth	Progression	(69, 70)
	HSF1**↑**	Downstream of TGF-β signaling YY1 enhances HSF1 expression and promotes proliferation and migration of breast cancer cells.	Progression	(95)

**Table 3:** YY1 Protein-protein interactions and their role in cancer progression/suppression.

**Interacting partner**	**Function of the interacting complex**	**Outcome/impact on cancer**	**References**
RelA	YY1 physically interacts with RelA and represses Bim gene in multiple myeloma	Progression of multiple myeloma	(41)
AR	YY1 interacts with AR and enhances the transcriptional activity of AR. induces prostate specific antigen expression	Prostate cancer progression	(97, 98)
P53, Mdm2	YY1 interacts with Mdm2 and p53 and facilitates Mdm2 mediated p53 ubiquitination and degradation	Breast cancer progression	(99)
P14^ARF^	Interaction of P14^ARF^ with YY1 impairs YY1 interaction with Mdm2 leading to p53 stabilization	Breast cancer suppression	(99)
PARP-1	YY1 interacts with PARP-1 and enhances PARP-1 enzymatic activity	Survival of cervical cancer cells	(100)
E1A	E1A binds to YY1-p300-HDAC3 complex and induces c-Myc expression	Tumor progression	(101)
*c-Myc*	c-MYC interaction with YY1 impairs YY1 transcriptional activity	Tumor progression/suppression??	(102)
E2F2/E2F3	YY1 interaction with E2F2/E2F3 results in enhanced CDC6 expression and cell cycle progression	Potential Tumor progression	(103)
PLK1	YY1-PLK1 interaction promotes cell division	Enhances growth of follicular lymphoma	(104)
P27	YY1 interacts with and enhances ubiquitination and degradation of p27	Tumor progression	(24)
HDAC2	YY1-HDAC2 complex represses miR-500a-5p expression and promote CRC cell proliferation	Progression of colorectal cancer	(45)
SFMBT2	SFMBT2 interacts with YY1 and represses HOXB13 gene and enhances DU145 prostate cancer cell survival	Prostate cancer progression	(63, 64)
	SFMBT2 interacts with YY1, represses MMP2, MMP3, MMP9 and inhibits LNCaP prostate cancer cell invasion and migration	Inhibition of prostate cancer cell invasion and migration.	
HDAC4	YY1 and HDAC4 complex represses HOXB13 gene in AR negative prostate cancer cells.	Prostate cancer progression	(49)
Activator protein 2 (AP-2)	Interaction of YY1 with AP-2 transcription factor induces ERBB2 expression in breast cancer cells.	Breast cancer progression	(69, 70)
ARAP1-AS1	YY1 causes up-regulation of ARAP1-AS1 expression which subsequently promotes cell migration, invasion, and ETM process in CRC via Wnt/β-catenin signaling mechanism.	CRC cancer progression	(105)
XAF1	YY1 inhibits XAF1 expression in prostate cancer cells lines through HDAC1 dependent mechanism and thereby induces cancer progression.	Prostate cancer progression	(91)
CARM1	CARM1 causes arginine methylation of YY1 and enhances its transcriptional activity.	Oral cancer progression	(106)
RelB	YY1-RelB complex promotes pro-inflammatory cytokines expression leading to glioma associated macrophage infiltration	Promotes glioma growth	(107)
Retinoblastoma (Rb)	Destabilizes YY1-DNA interaction to inhibit YY1 mediated gene activation	Potential tumor suppression	(65)
SYK(L)	YY1-SYK(L) interaction suppress epithelial-to-mesenchymal transition (EMT) by inhibiting SNAI2 transcription	EMT inhibition	(108)
P300 & AP-1	Induces expression of tumor suppressor HLJ1	Suppression of lung cancer	(85)

## Breast Cancer

Several studies demonstrated YY1 overexpression in breast cancer cell lines and primary tumors leading to tumor promotion (24, 69, 70, 113). Mechanistically, YY1 has been shown to coordinate with AP2 to induce the oncogene Erbb2 in breast cancer cells (69, 70) leading to tumor aggressiveness. In line with this, depletion of YY1 led to impaired tumor formation, colony formation as well as migration whereas overexpression of YY1 resulted in the opposite effect (24). Further, YY1 has been shown to repress the expression of cell cycle inhibitor p27 and also has been shown to physically interact with p27 and cause its ubiquitination via Skp2 leading to unopposed growth of breast cancer cells (24). Recently, Yang et al. showed that FAM3C upregulates YY1, which in turn induces HSF1 expression leading to proliferation and migration of breast cancer cells through the Akt-Cyclin D1 pathway (95). Further, they showed that FAM3C-YY1-HSF1 signaling axis is essential for TGFβ induced proliferation and migration of breast cancer cells. Hence, targeting the FAM3C-YY1-HSF1 signaling axis is an effective strategy for treating TGFβ-dependent breast cancer (95).

**Figure 2:**
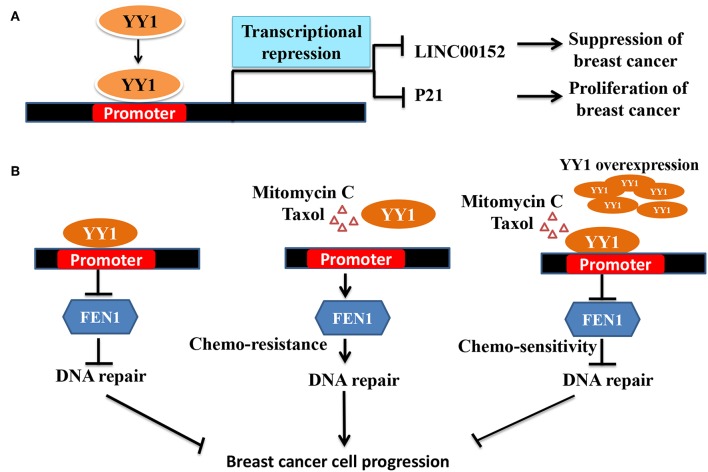
Schematic representation of YY1 mediated transcriptional repression leading breaset cancer suppression/progression. **(A)** YY1 binds to promoter region of LINC00152 and P21, and represses their expression levels, which lead to tumor suppression and progression, respectively. **(B)** In some groups of breast cancer patients YY1 was shown to function as tumor suppressor. FEN1 levels have been reported to be high in breast cancer cells and helps in tumor growth (115). Owing to its important role in DNA damage repair and replication, targeting FEN1 was shown to inhibit tumor growth. YY1 was shown to bind to FEN1 promoter and repress FEN1 expression leading to better survival of cancer patients. Chemotherapeutic agents including mitomyin C and Taxol reduce YY1 expression leading to elevated levels of FEN1 and drug resistance and tumor growth. However, ectopic expression of YY1 can restore the sensitization of breast cancer cells to chemotherapeutic agents by repressing FEN1.

Interestingly, contradictory results by Lee et al. (23) showed that YY1 plays a tumor suppressive role in breast cancer. Lee et al., showed that YY1 is highly expressed in normal breast tissue compared to breast cancer tissue and that YY1 positively regulates expression of BRCA1, a tumor suppressor. In line with this finding, depletion of YY1 in MDA-MB-231, cells has been shown to express low levels of BRCA1 and overexpression of YY1 led to increased BRCA1 and tumor suppression (23). However, the mechanism of YY1 down regulation in breast cancer was not clear from this study. Similarly, in another study by Lieberthal et al. (114) showed that low levels of YY1 contributes to invasive phenotype of breast cancer cells and that overexpression of YY1 suppressed HS578T breast cancer cells migration by regulating the expression of HP1alpha, which might play a role in the invasiveness of breast cancer cells. In a recent report, Shen et al. (78) have further reported the tumor suppressive role of YY1 in triple negative breast cancer (TNBC) wherein YY1 was shown to exhibit transcriptional repressive function to repress LINC00152 expression (Figure 2A). They further showed that LINC00152 targets the tumor suppressor PTEN to proteasome dependent degradation. Hence, YY1-dependent repression of LINC00152 expression leads to elevated PTEN levels and suggested that YY1/LINC00152/PTEN axis plays an important tumor suppressive role in triple negative breast cancer (78). In contrast Liang et al. (116) showed that YY1 promotes TNBC by suppressing the transcription of miR-5590, which upon overexpression has been shown to inhibit TNBC growth. YY1 was shown to directly bind to miR-5590 promoter and represses it expression. Moreover, overexpression of YY1 was shown to counter the negative impact of mir-5590 overexpression on TNBC growth (116). The transcriptional repressive function of YY1 was further shown to result in suppression of breast cancer growth by direct repression of FEN1 gene (Figure 2B) (79) and indirect repression of FEN1 through inducing miR-140 expression, which was shown to target FEN1 3′UTR (117). FEN1, which is a key player in DNA replication, repair as well as alleviating the replication stress, is important for tumor growth. Hence, YY1-dependent repression of FEN1 leads to sensitization of breast cancer cells to DNA-damaging agents (79, 117).

Collectively, the two sides of YY1 i.e., transcriptional activation and repression were shown to bring out the two sides of YY1 in breast cancer i.e., tumor promotion vs. tumor repression. YY1-dependent transcriptional activation and repression of ERBB2 and p27 respectively, leads to tumor promotion. On the other hand, YY1-dependent positive regulation of BRCA1 and HP1alpha results in tumor suppression and reduced invasiveness. Negative regulation of LINC00152 and FEN1 by YY1 leads to tumor suppression.

## Pancreatic Cancer

**Figure 3:**
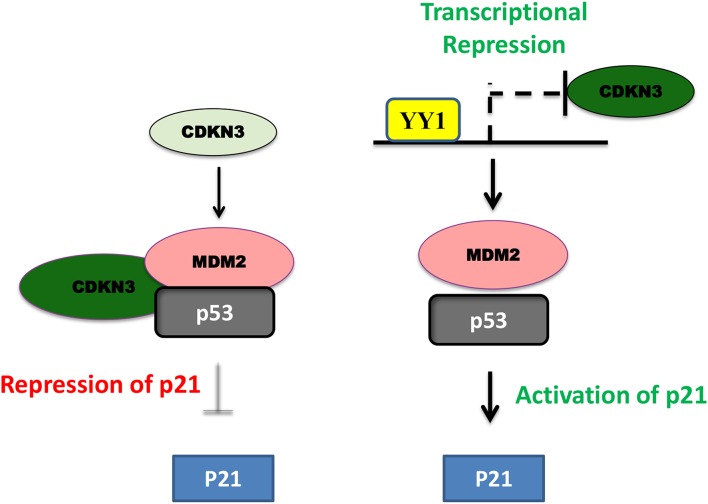
Schematic representation of YY1-CDKN3-MDM2 interaction and its functional significance in cancer. In pancreatic cancer, CDKN3 was shown to form a complex with MDM2-p53 and inhibit p53 dependent upregulation of p21 leading to tumor cell proliferation. YY1 was shown to transcriptionally repress CDKN3 and thereby promote p53 dependent p21 expression and impair in pancreatic cancer cell proliferation.

In pancreatic cancer YY1 largely has a tumor suppressive role and its expression relative to other tumor types, is less in pancreatic cancer (https://www.proteinatlas.org/). In line with this, high levels of YY1 expression in pancreatic cancer has been shown to have better clinical outcome (22). A recent study by Chen et al. (84) shown that overexpression of YY1 inhibits pancreatic cancer growth both *in vitro* and *in vivo*. YY1 was shown to bind to Feline sarcoma-related (FER) promoter and repress its expression; where FER has been shown to induce the phosphorylation of STAT3, which in turn enhances the expression of MMP2 and cancer progression. Collectively, YY1 inhibits FER expression, which in turn results in impaired STAT3-MMP2 pathway leading to suppression of pancreatic cancer growth. Zhang et al. have further shown that YY1 prevents invasive metastasis of pancreatic ductal adenocarcinoma (PDAC) by downregulating the expression of MMP10 in a MUC4/ErbB2/p38/MEF2C dependent manner indicating the tumor suppressive role of YY1 in pancreatic cancer. Recently, Liu et al. (80) have shown that over-expression/knockdown of YY1 inhibits/stimulates the proliferation and infiltration of PDAC cells. They found that YY1 binds to CDKN3 promoter and represses its expression and that CDKN3 was shown to interact with MDM2-p53 complex and inhibit the expression of cell cycle inhibitor p21 (Figure 3). Thus, by blocking CDKN3 expression YY1 promotes p21 expression and tumor suppression.

The tumor suppressive role of YY1 in PDAC further came into light by the observation that YY1 exhibits transcriptional repression of the tumor promoting lncRNA SOX2OT and subsequent down regulation of the cancer stem cell marker SOX2 (81). It has been shown that lower expression of SOX2OT had better outcome in PDAC. In addition to regulating the lncRNA SOX2OT, YY1 was also shown to repress miR-30a, in pancreatic cancer cells and thereby suppress pancreatic tumor growth presumably by modulating autophagy (82). In contrast to its transcriptional repressive function, YY1 as a transcriptional activator was shown to induce the expression of the pro-apoptotic BAX gene and induce apoptosis of pancreatic cancer cells and prevent tumor growth (83). Collectively, while YY1 largely plays a tumor suppressive role in pancreatic cancer, it is not clear as to how YY1 levels were maintained relatively low in pancreatic cancer compared to other cancer tissues.

## Colon Cancer

YY1 is largely shown to be pro-tumorigenic in colon cancer. It has recently been shown that a unique type of protein modification, O-GlcNAcylation plays an important role in modulating protein stability and function, and was shown to exhibit important pro-tumorigenic role in colorectal cancer (CRC) (88). Importantly, O-GlcNacylation may perhaps be considered as a new cancer hall mark (118). While YY1 is elevated in CRC, interestingly, YY1 has been shown to be O-GlcNAcylated at Thr-236 by O-GlcNAc transferase (OGT), leading to increased stability and transcriptional activity of YY1 (88). This in turn results in YY1-dependent induction in the expression of metabolite transporter SLC22A15, and an enzyme involved in melatonin synthesis, AANAT. In line with this, Zhu et al., have shown that over expression of SLC22A15 and AANAT increases the survival and proliferation of CRC cells and their depletion results in the opposite outcome (88). Thus, targeting YY1 O-GlcNAcylation could serve as an attractive therapeutic strategy for colon cancer treatment. In addition to being O-GlcNacylated, YY1 expression is upregulated in CRC and is linked to tumor proliferation, high grade metastasis and drug resistance. It has been shown that corticotropin releasing hormone receptor 2 and urocortin CRHR2/Unc2 signaling is downregulated in CRC cells and that down regulation of CRHR2 is essential to maintain high levels of YY1 in CRC. CRHR2/Unc2 signaling results in elevation of miR-7, which in turn targets YY1 leading to elevated Fas expression and death in response to agonistic Fas antibody. Due to the low levels of CRHR2 in CRC cells, YY1 levels are high, which in turn represses Fas expression resulting in enhanced CRC cell survival (86, 119). In line with this, it has been shown that YY1 negatively regulates Fas gene (120). In addition to miR-7, miR-215 has also been shown to target YY1 leading to reduction in YY1 levels. Owing to the pro-tumorigenic role of YY1 in CRC, both miR-7 and miR-215 were shown to be down regulated in CRC cells (121, 122). Further, YY1 was also shown to repress the tumor suppressor miR-500a-5p and promotes CRC tumor progression in a p300/YY1/HDAC2 dependent manner (45). These authors also found that YY1 expression was elevated in CRC and its expression was inversely proportional to miR-500a-5p expression. Another recent study by Yaqun et al. (105), has also revealed elevated YY1 expression in CRC. YY1 enhances transcription of lncRNA ARAP1 antisense RNA 1 (ARAP1-AS1), which is correlated with increased cell migration, invasion and EMT process in CRC via Wnt/β-catenin signaling mechanism. In addition to promoting the Wnt signaling via ARAP-AS1 expression, YY1 also promotes Wnt pathway in CRC by directly repressing the dominant negative isoform of LEF-1 (DN-LEF1), which is a inhibitor of Wnt signaling (87). Collectively, in colon cancer, YY1 is largely a tumor promoter and an attractive drug target.

## Lung Cancer

YY1 expression has been shown to be elevated in lung cancer tissues and YY1 has been shown to play a key pro-tumorigenic role in lung cancer (90). Among the signaling pathways associated with lung cancer progression, NF-κB, IL-13 and the PI3K/AKT pathways were shown to upregulate YY1 (41, 123, 124). While NF-κB directly activates the transcription of YY1, IL13 signaling upregulates YY1 in a AKT dependent manner (125). YY1 plays a key role in normal lung development (126) and its overexpression leads to cancer progression by positive regulation of many genes including long noncoding RNA plasmacytoma variant translocation 1 (lncRNA-PVT1) (90), miR-1260b as well as COX2 (127). YY1 has also been implicated in epithelial mesenchymal transition (EMT) by inducing expression of SLUG (108). However, the long form of spleen tyrosine kinase (SYK-L), was shown to directly interact with YY1 and interfere with the expression of SLUG and thereby the SYK-L functions as a tumor suppressor and reduce the invasiveness of lung cancer cells (108). Among other factors that might interfere with YY1 function in lung cancer cells, miR-29a and miR-186 have received significant attention. While both miR-29a and miR-186 were shown to down regulate YY1, YY1 in turn was shown to repress miR-186 and promote lung tumorigenesis (128, 129).

Interestingly, while most studies on YY1 in lung cancer revealed a pro-tumorigenic role, Wang et al. (85) reported that YY1 coordinates with AP1 to induce the expression of tumor suppressor chaperone HLJ1 in human lung adenocarcinoma cells and was shown to reduce the invasiveness of these cells. Further, the tumor suppressive role of YY1 in lung cancer was also evidenced by the finding that YY1 binds to the promoter of tumor suppressor microRNA-520c-3p and enhances its expression. Enhanced expression of miR-520c-3p inhibits tumor progression by negatively regulating AKT1 and AKT2 (130). Such contrasting roles of YY1 both as a tumor promoter and tumor suppressor is puzzling and further work is required to gain a complete mechanistic insight into these opposing functions of YY1.

## Prostate Cancer

Similar to other cancers, YY1 has been reported to be overexpressed in prostate cancer also and has been suggested to play essential role in proliferation, invasion, metastasis and drug resistance (131). Mechanistically, to promote prostate cancer growth, YY1 has been shown to repress the expression of X-linked inhibitor of apoptosis (XIAP) associated factor-1 (XAF1), a tumor suppressor in prostate cancer cells (91). It was shown that YY1 binds to XAF1 promoter and thereby inhibits its expression in prostate cancer cell lines in a HDAC1 dependent mechanism (91). In addition to repression of regular genes, YY1 has also been shown to repress tumor suppressive miR-146a in prostate cancer cells upon interacting with EZH2 and thereby promotes prostate tumor growth (111). Further, YY1 also contributes to the metastatic potential of the prostate cancer cells by mediating transcriptional repression of heterogeneous nuclear ribonucleoprotein M (hnRNPM), which is an inhibitor of migration and invasion of prostate cancer cells (132). Further, like in the case of lung cancer, miR-186 was shown to be down-regulated in prostate cancer cells and its overexpression decreases cell proliferation and tumor growth by targeting YY1 (133). However, as mentioned above, YY1 also represses miR-186 and hence might promote prostate tumorigenesis. Likewise, an inverse correlation in the expression of YY1 and miR-146a has been reported in prostate cancer (111) as explained above.

YY1 has also been linked to regulate tumor cell metabolism. Interestingly, while in colon cancer cells YY1 has been linked to promoting the Warburg effect (89), in prostate cancer cells, Park et al. (134) showed that YY1 regulates genes associated with mitochondrial energy metabolism (i.e., Krebs cycle and electron transport chain). In line with this finding, interestingly, while most tumors exhibit an addiction to the aerobic glycolysis (Warburg effect), early prostate cancers appear not to rely on the Warburg phenomenon and other energy metabolic pathways seem to promote early prostate tumor growth (135).

While, YY1 largely acted as a tumor promoter in prostate cancer, upon interacting with SFMBT2 (Scm-like with four mbt domains 2), YY1 acts both as a tumor promoter and suppressor in prostate cancer. In case of DU145 cells SFMBT2 interacts with YY1 and represses HOXB13 gene and enhances cell survival. In case of LNCaP, SFMBT2 interacts with YY1 and represses MMP2, MMP3, and MMP9 and inhibits invasion and migration of prostate cancer cells, and illustrating both pro and anti-role of YY1 in prostate cancer growth.

## Cervical Cancer

Human papilloma virus (HPV) infection has been the major cause of cervical cancer. Baritaki et al. (136) reported that YY1 was overexpressed in cervical cancer cells from patients infected with HPV-18 or HPV-16. Further, it has been shown that depletion of YY1 in HPV positive HeLa cells led to increased p53 expression and apoptosis (137). This study has further, implicated that elevated YY1 levels contributes to drug resistance in cervical cancer. Specifically, treatment of HeLa cells with arsenic trioxide (As2O3) was shown to reduce YY1 expression and correlated with increased apoptosis. It appears from these studies that YY1 limits p53 expression and protects cervical cancer cells from apoptosis. Mechanistically, YY1 plays other key roles in promoting cervical cancer progression by repressing miR-181 and modulating the expression of E-Cadherin and HPV E6 oncoprotein (138, 139). miR181a was previously shown to inhibit cervical cancer growth by targeting the unfolded protein response regulator GRP78 (140). Zhou et al. (139) have found that while miR-181 targets YY1 and inhibits cervical cancer growth, YY1 represses miR-181 expression and promotes tumor growth. Also, YY1 appear to positively regulate the oncogene HPV E6 and negatively regulate E-Cadherin and thereby promote tumor growth and metastasis (138).

## Thyroid Cancer

Compared to other cancers, YY1 role in thyroid cancer has not been well-explored. Recently, Arribas et al. (141) demonstrated that both YY1 mRNA and protein levels were over-expressed in thyroid cancer cells as compared to their adjacent normal cells. Moreover, immuno-histochemical of analysis of cancer tissue microarrays revealed that YY1 levels were highly expressed in papillary thyroid cancer (PTC) compared to follicular thyroid cancer (FTC). While the mechanistic details on how YY1 might contribute to the progression of thyroid cancer is not clear, it has recently been shown that two micro RNAs, miR-544, and miR-141-3p inhibit thyroid cancer growth by targeting YY1 (142, 143). Further studies on the role of YY1 in thyroid cancer are needed to gain mechanistic understanding of how YY1 promotes thyroid tumorigenesis.

## Liver Cancer

YY1 has been implicated in hepatocellular carcinoma (HCC). Reports showed that YY1 enhances HCC by regulating expression of several genes (144). While it was previously documented that YY1 coordinates with HDACs to regulate diverse genes, Dong et al. (144) showed that YY1 directly binds to HDAC1 promoter and induces its expression and there by YY1 attenuates the sensitivity of HCC cells to HDAC inhibitors. In another study, Tsang et al. (145) showed that YY1 and EZH2 levels were upregulated in HCC that correlated with poor survival of HCC patients. Further, they showed that YY1 and EZH2 mediate histone H3 lysine 27 trimethylation to silence several tumor suppressive miRNAs. Overexpressed YY1 recruits EZH2 for H3K27me3-mediated down-regulation of miR-9 thereby enhancing NF-κB activation and pro-tumorigenic phenotypes (145).

TCGA data set shows that high YY1 and QKI expression leads to shortend survival of HCC patients. Further, they showed that Yin-Yang 1 (YY1)/p65/p300 complex activates the Quaking (QKI) gene expression which is required to promote metastasis and malignancy of HCC. QKI control expression of circRNA including circ-0008150, circ-0007821 to induce EMT in HCC (146). Further, Chiung-Yuan Ko et al. (147) showed that YY1 upon interacting with suppressor of Zeste 12 (SUZ12) recruits polycomb repressive complex to the promoter of the tumor suppressor gene CEBPD (148) to repress its expression in HCC and thereby promotes HCC growth.

Surprisingly, in another study by Zhang et al. (149) hepatitis B virus (HBV) protein was shown to upregulate the tumor suppressor HLJ1 expression via YY1. This raises a question as to whether YY1 functions as a tumor suppressor in the case of HBV mediated liver cancer.

## Hematological Malignancies

**Figure 4:**
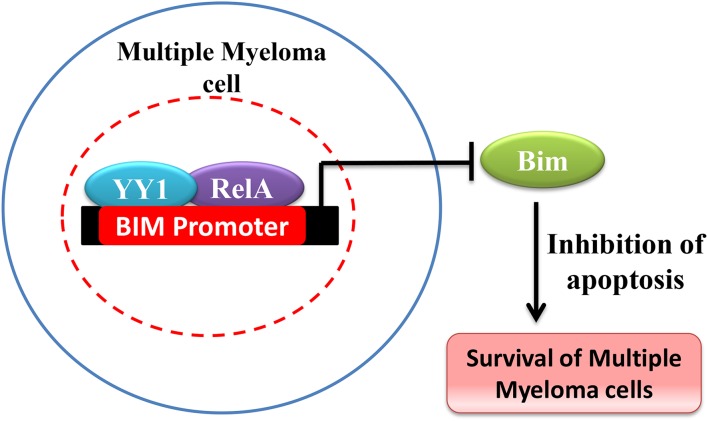
Schematic representation of Bim repression by YY1-RelA complex. In Multiple Myeloma YY1 forms a complex with RelA and YY1-RelA complex represses Bim and promotes multiple myeloma cell survival and growth.

YY1 has been shown to be over expressed and promote cancer in hematological malignancies (150). For instance in Multiple Myeloma (MM), Potluri et al. have shown that YY1 is highly expressed and is largely nuclear localized in MM cells (41). While YY1 expression is NF-κB dependent in MM, interestingly, YY1 forms a transcriptionally repressive complex with RelA and the YY1-RelA complex plays a key role in the survival of MM cells (41). The YY1-RelA complex was shown to directly bind and repress the pro-apoptotic Bim gene in MM cells and depletion of YY1 or RelA completely impaired the colony forming ability of MM progenitor cells, suggesting that RelA-YY1 complex formation (Figure 4) is absolutely essential for the survival and growth of MM cancer cells (41). However, the mechanism by which YY1-RelA represses Bim gene needs to be further elucidated. Recently, Huerta-Yepez et al., showed that YY1 is overexpressed in bone marrow derived multiple myeloma cells and its expression is correlated with poor prognosis and that depletion of YY1 in drug resistant MM cells sensitized them to bortezomib induced apoptosis (151) and it is likely that YY1 might also interact with factors other than RelA to promote MM growth. While YY1 was shown to constitutively form the repressive YY1-RelA complex in MM, the signaling pathway/s involved in this remained elusive and whether YY1-RelA complex plays a pro-tumorigenic role in other cancer models has not been explored. Thus, understanding of YY1-RelA complex formation, and its association with different cancers would substantially help in the design of novel and effective therapeutic approaches and drug development.

YY1 expression has also been shown to be highly elevated in Burkitt's lymphoma and diffuse large B-cell lymphoma (DLBCL) as compared to normal cell B cells, and its expression levels links with B-cell transformation and tumor progression (152). In line with this inhibition of YY1 has been shown to sensitize NHL cells to TRAIL mediated apoptosis (153). Further, the oncoprotein PLK1 interacts with and phosphorylates YY1 in follicular lymphoma cells. Phosphorylation of YY1 by PLK1 activates YY1 and leads to increased cell proliferation (104). Recently Morales-Martinez et al., showed that YY1 regulates Krüppel-Like Factor 4 (KLF4) protein transcription through binding to its promoter region in lymphoma cell lines (93). Interestingly, while KLF4 was shown to be a tumor suppressor in NHL (154), Morales-Martinez et al., suggested that YY1 positively regulates KLF4 expression and that YY1 expression in NHL cells correlates with KLF4 expression (93). This raises a question whether KLF4 may also promote NHL in cases of combined hyper expression of YY1 and KLF4.

In addition to lymphoma and myeloma, YY1 has also been shown to play an important role in promoting acute myeloid leukemia (AML). The SDF-1a/CXCR4 signaling has been shown to upregulate YY1 in AML cells in which YY1 downregulates the tumor suppressor microrna miR-let-7a and upregulates MYC and BCLXL to promote AML growth (94). In a recent article by Antonio-Andres et al. (155), YY1 has been shown to have a pro-tumorigenic role and enhance chemo-resistance in acute lymphoblastic leukemia (ALL) by inducing the expression of multi-drug resistance 1 gene (MDR1), which is necessary for ALL chemo-resistance. In line with this, these authors also have reported high expression levels of both YY1 and MDR-1 in ALL patients. Thus, in hematological malignancies, YY1 largely plays a pro-tumorigenic role.

## Melanoma

YY1 expression was shown to be elevated in melanoma patient samples and its expression levels were shown to increase as tumor progresses to advanced stages (109). In this report, YY1 has been shown to be essential for proliferation, survival and metastasis of melanoma cells. Interestingly, while miR-9 was shown to be an inhibitor of melanoma metastasis by downregulating the NF-κB1-Snail pathway, YY1 was shown to suppress miR-9 transcription and hence promote metastatic potential of melanoma cells (109, 156). In addition to repressing miR-9, YY1 also has been shown to directly bind to *snail* gene enhancer region and activate *snail* transcription and thus promote EMT and metastasis of melanoma cells (157). Interestingly, while autophagy may either promote or suppress tumor growth, in melanoma cells, YY1 was shown to coordinate with Transcription factor EB (TFEB) and regulate genes involved in autophagy and lysosomal biogenesis (158). In this report, it was speculated that YY1 mediated autophagy might provide resistance to BRAF inhibitor vemurafenib, and this notion was supported by the observation that suppression of YY1 sensitizes melanoma cells to vemurafenib both *in vitro* and *in vivo* (158). While, a direct link between enhanced autophagy and resistance to vemurafenib is not very clear from this report, autophagy inhibition has indeed been shown to sensitize cancer cells to vemurafenib (159, 160). In a more recent report, the role of YY1 in melanoma tumorigenesis has been directly shown using melanoma mouse model, in which ablation of one YY1 allele impaired melanoma tumor growth by regulating metabolism and protein translation (75).

## Glioblastoma

YY1 expression has been shown to be elevated in glioma tumors and its expression has been attributed to tumor progression (161). Experimentally, YY1 has been shown to contribute to proliferation of glioma cells, as evidenced by impaired proliferation and enhanced p53 expression upon YY1 depletion in glioma cells (92). In this report it has been shown that miR-218 targets YY1 leading to its downregulation and impaired glioma cell proliferation. Interestingly, in glioblastoma multiforme (GBM) the NF-κB family member RelB was shown to interact with YY1 and promote expression of GBM specific genes as well as pro-inflammatory cytokines leading to infiltration of glioma associated macrophages and thus tumor progression (43). Further, YY1 was shown to confer resistance to drugs such as cisplatin in GBM (107). These results clearly indicate that YY1 plays a key role in glioblastoma cell growth and chemo resistance. Collectively, in glioma, YY1 appears to act as a tumor promoter.

**Figure 5:**
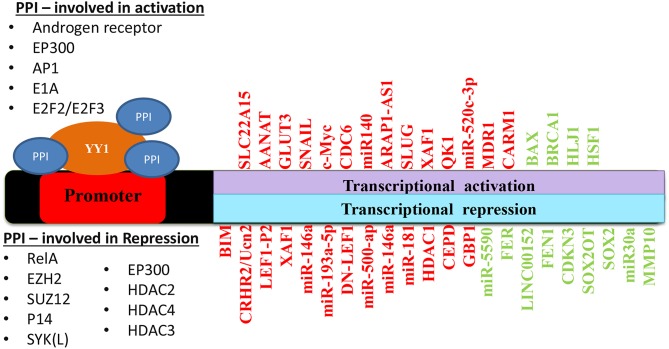
Schematic representation of YY1 protein-protein interactions involved in transcriptional activation and repression of various genes in different cancers. Red color represents an outcome on tumor progression and green color represents tumor suppression.

Taken together, depending on its interacting partners and genes that YY1 regulate, it acts as a tumor promoter or suppressor. A list of YY1 interacting partners regulating gene expression/repression in the context of cancer growth has been illustrated in Figure 5. Also, YY1 expression patterns observed in primary tumor tissues, various cancer cell lines as well as animal models for YY1 role in cancer are listed in Table 4.

**Table 4:** YY1 expression analysis in patient derived tumor samples, cancer cell lines, and xenografts.

**Cancer type**	**Cell lines**	**Xenograft**	**Patient data**	**YY1 expression in cancer cell lines**	**Database**	**Clinical outcome**	**References**
Breast	MCF-7, MDA-MB-231, SK-BR-3, ZR-75-1, BT-474	Knockdown	Tissue Microarray-IHC-High expression	High	-	Tumor progression	(24)
	MCF7 and MDA-MB-231, BT474	-	-	High	-	Tumor progression	(70)
	-	-	Meta-analysis–High expression	-	Meta-analysis	Promotion of metastasis	(113)
	MDA-MB-231	Ectopic YY1 overexpression	Tissue array-IHC— Low	-	Oncomine	Tumor suppression	(23)
	HS578t	-	-	Low	-	Low YY1 levels were shown to promote invasiveness	(114)
Pancreatic	**HPAC, SW1990**, BXPC-3, CFPAC-1, PANC-1, COLO-357	Knockdown	Tissues—IHC—High expression	High	-	Inhibits cell migration and tumor proliferation	(22)
	BxPC-3, COLO-357, CFPAC-1, PANC-1 and HPNE (non-cancer)	Knockdown	-	High	-	Inhibits migration and Invasion	(84)
	PANC-1 cells and BXPC-3	Ectopic YY1 over expression	mRNA analysis—High expression			Inhibits tumor progression	(80, 83)
Colon	HCT116, DLD1 and LOVO	Knockdown	Tissue array-IHC— High expression	High	-	Inhibits apoptosis and promotes tumor growth.	(121, 122)
Lung-	A549, 95D, HCC827, and NCI-H165	Knockdown	Tissue array—IHC—High expression	High	-	Inhibits apoptosis, increases proliferation and promotes tumor growth	(90)
	A549 HCC827	-	Tissue array—mRNA- High expression	High		Inhibits apoptosis and promotes invasion and migration.	(128)
	CL1-5	-	-	Ectopic YY11 overexpression	-	Inhibit cell invasion. Potential tumor suppression	(85)
Prostate	**-**	-	-	High	Oncomine	Tumor promotion	(131)
	LNCaP, C4-2, PC-3, DU145	-	Tissues—IHC—High expression	High	Oncomine	Enhances EMT	(132)
Cervical	**-**	-	Tissues—mRNA analysis and IHC— High expression	-		Correlates with tumor progression	(136)
	**Hela**	-	Tissues—Western blot—High expression	High	-	Inhibits apoptosis and promotes tumor growth	(137)
	**-**		Tissue- IHC—High expression	High	-	Correlates with tumor progression	(138)
Thyroid	TPC-1, BCPAP, ML1, WRO, CGTH, FRO, 8305, 8505, CAL-62.	-	Tissues- IHC - Western Blotting - mRNA analysis—High expression	-	-	Correlates with tumor progression	(141)
Liver	SMMC-7721, HUH-7, BEL-7404, HEPG2	Knockdown	Tissues- mRNA analysis—High expression	High		Decreases sensitivity to HDAC inhibitors—Tumor progression	(144)
	HEP3B, HKCI-8	Knockdown	Tissues—IHC—High expression	HIgh	-	Inhibits apoptosis and promotes tumor growth	(145)
Hematological malignancies	KMM1, JJN3, OCI-My1, H929 (Multiple Myeloma)	Knockdown	Patient derived Primary cells—Western blotting—High expression	High	-	Inhibits apoptosis and promotes tumor growth	(41)
	MM1s, 8266, IM-9, U266 (Multiple Myeloma)		Primary cells— western blot	High	Oncomine—High expression	Inhibits apoptosis and promotes tumor progression. Knockdown sensitizes cells to Bortezomib induced apoptosis	(151)
	RAJI, RAMOS, DAUDI (Lymphoma)	-	Western blot	High	Microarray dataset—High expression	Correlates with tumor progression	(93, 152)
	RS4;11	-	Primary ALL cells—High expression	High		Tumor progression and decreased patient survival	(155)
Melanoma	WM852, WM1791C, WMB, WM209		Tissues-mRNA—High expression	-	-	Promotes tumor growth	(109)
	A375	Knockdown	-	-	-	Decreases sensitivity to vemurafenib and promote tumor progression by modulating autophagy	(158)
		Conditional deletion in melanoma mouse model				YY1 deletion impairs melanoma growth	(75)
Gliobastoma	U251MG, LN229	-	-	High expression in cisplatin resistant cells	-	Promote glioma tumor progression by providing resistance to cisplatin	(107)
	**-**	-	Tissue mRNA—High expression			Promotes tumor growth	(161)

## Targeting YY1 in Cancer

Although YY1 has been shown to have both tumor promoting and tumor suppressive roles in different cancers, it largely plays a role as a tumor promoter in many cancers. Owing to its central role in tumor progression, YY1 is an attractive drug target. Several recent reports have indicated targeting YY1 by various drugs in different cancer models. While some of those drugs such as Nitric Oxide donors directly acted on YY1 to prevent its DNA binding, some drugs acted indirectly by reducing the levels of YY1 expression. A list of drugs and their effect on YY1 and the impact on cancer growth is provided in Table 5.

**Table 5:** YY1 reported inhibitors and their inhibition mechanism in different cancers.

**S. no**	**Name of inhibitor**	**Inhibition mode**	**Outcome**	**References**
1	NO donors: S-nitroso-N-acetylpenicillamine (SNAP) and DEA-NONOate	Promotes Fas gene expression by impairing YY1 binding to the Fas gene promoter.	Inhibition of ovarian cancer	(120)
	DETANONOate	Inhibits NF-κB and its down-stream target snail and YY1. Also, directly S-nitrosylates and deactivates YY1	Inhibition of prostate cancer	(162)
2	Betulinic Acid (BA)	BA targets YY1 expression levels through cannabinoid receptor (CB) dependent mechanism and thereby inhibits YY1 expression levels.	Inhibition of cell growth and induction of apoptosis in breast cancer	(163)
4	Rituximab	Inhibits NF-κB and thereby reduces YY1 mRNA and protein levels.	Inhibition of B-cell non-Hodgkin lymphoma	(164)
5	Galiximab	Inhibits NF-κB activity and thereby reduces YY1 expression levels.	Sensitizes the B-NHL cells to TRAIL mediated apoptosis.	(165)
6	Proteasome inhibitor (Salinosporamide A-NPI-0052)	Inhibits NF-κB and thereby reduces YY1 mRNA and protein levels.	Sensitizes Burkitt's lymphoma cells and prostate cancer cells to TRAIL mediated apoptosis.	(166)
7	onco-protein binding (OPB) domain of YY1 (aa G201- S226)	OBP peptide disrupts YY1 interaction with other oncogenic proteins including AKT and thereby prevents cancer growth.	Hinders breast cancer progression	(167)
8	MicroRNAs:	All microRNAs (miRNAs) mentioned here directly bind to YY1 3'UTRs and thereby inhibits its expression levels in different tumors as indicated		
	miR-141-3p		Inhibition of human papillary thyroid cancer	(142)
	miR-29a		Inhibition of lung cancer	(168)
	miR-186		Inhibition of Lung and prostate cancer	(128)
	miR-181		Inhibition of cervical cancer	(139)
	miR-7		Inhibition of colorectal cancer	(122)
	miR-544		Inhibition of thyroid cancer	(143)
	miR-218		Inhibition of human glioma cells	(92)

## Conclusion

While YY1 has been extensively studied for its role in gene regulation (both activation and repression) and cancer, the mechanism by which YY1 contributes to tumor growth differs in different cancers. Importantly, its role both as a tumor promoter and tumor suppressor is of significant interest. Although YY1 plays a tumor promoting role in many cancers, it largely plays a tumor suppressive role in pancreatic cancer. In this context, it remains to be tested whether the YY1 interactome is significantly different in pancreatic cancer compared to other cancers. Interestingly, in the case of breast cancer, it is quite puzzling that YY1 functions as both tumor promoter and tumor suppressor. Different groups observed different outcomes for YY1 function in breast cancer and it is difficult understand how YY1 plays such opposing roles within a given cancer type. In this context, it is perhaps likely that specific interacting partners of YY1 might be either missing or overexpressed within different breast cancer patients and hence the opposing outcome. At the end, it is reasonable to argue that the specific nature of YY1 interactome in different cancers would govern the function of YY1 leading to either tumor suppression or tumor promotion. Likewise, the YY1 interactome would govern its function as transcriptional repressor / activator. Hence, a detailed picture of YY1 interacting partners from different cancer models would help us understand the biology of YY1 in cancer to a greater detail, which would eventually help us develop novel therapeutic strategies for cancer cure that are centered around targeting YY1.

## Author Contributions

This is a originally written review on YY1 by SS. Significant effort in writing has also been put up by SK. SV has written and corrected the manuscript.

### Conflict of Interest

The authors declare that the research was conducted in the absence of any commercial or financial relationships that could be construed as a potential conflict of interest.
